# Purification and characterization of laccase from *Marasmius* species BBKAV79 and effective decolorization of selected textile dyes

**DOI:** 10.1007/s13205-016-0504-9

**Published:** 2016-09-02

**Authors:** A. B. Vantamuri, B. B. Kaliwal

**Affiliations:** 1 Department of Studies and Research in Biotechnology and Microbiology, Karnatak University, Dharwad, 580003 India; 2 Department of Studies and Research in Biotechnology and Microbiology, Davangere University, Davangere, 577 002 India

**Keywords:** Decolorization, Laccase, *Marasmius* sp. BBKAV79, Purification

## Abstract

A novel laccase-producing white-rot fungus, *Marasmius* sp. BBKAV79 (Genbank Accession Number-KP455496, KP455497), was isolated and subjected to purification, characterization and dye decolorization study. The purified enzyme was obtained with a specific activity of 0.226 U mg^−1^ protein and a final yield of 13.5 %. The enzyme was found to be a monomeric protein with a molecular mass of ~75 kDa as estimated by non-denaturing polyacrylamide gel electrophoresis (PAGE) and further confirmed with zymogram analysis. The optimal pH and temperature of the laccase was recorded to be 5.5 and 40 °C, respectively. The metal ions Hg^2+^ and Ag^+^ were found to drastically inhibit the activity of laccase at the rate of 96.6 and 96.5 %, respectively. Nevertheless, Fe^3+^ was found to inhibit laccase activity at 40 %. Phenylmethanesulfonyl fluoride (PMSF) strongly inhibited the laccase activity, and additives viz, sodium dodecyl sulfate (SDS), hydrogen peroxide (H_2_O_2_) and sodium chloride (NaCl) were known to follow the earlier pattern of enzyme inhibition. The values of kinetic parameters *K*_m_ and *V*_max_ for purified laccase were noted at 3.03 mM and 5 μmol min^−1^, respectively, for guaiacol as substrate. The textile dyes were decolorized at a range of 72–76 % and 88–93 % when treated with *Marasmius* sp. BBKAV79 and purified laccase, respectively. Based on the outcome of the present investigation, it could be, therefore, inferred that laccase isolated from *Marasmius* sp. BBKAV79 effectively decolorizes the textile dyes; however, the metal ions Hg^2+^, Ag^+^ and Fe^3+^ and agents like PMSF, SDS, H_2_O_2_ and NaCl pose an effective inhibitory potential under specified physicochemical conditions.

## Introduction

Relentless production, utilization and dumping of synthetic organic chemicals has contributed to environmental pollution globally (David and Kartheek 2015; Malaja et al. 2014). Synthetic dyes are one such class of chemicals that are broadly used in wide range of industries including textile, paper, printing, cosmetics and pharmaceuticals (Vinodhkumar et al. 2013). There are many structural varieties of dyes with respect to the type of chromophore, viz azo, anthraquinone, acridine, arylmethane, cyanine, phthalocyanine, nitro, nitroso, quinone-imine, thiazole or xanthene dyes. It is estimated that 10–15 % of the dyes are lost in the effluent during dyeing process (Houria and Oualid 2009). Many synthetic dyes are difficult to decolorize due to their complex structure. Decolorization of textile dye effluent does not occur when treated aerobically by municipal sewage systems (Willmott et al. 1998). Brightly colored, water-soluble, reactive and acid dyes are the most problematic, as they tend to pass through conventional treatment systems unaffected (Willmott et al. 1998). Color can be removed from effluent by chemical and physical methods including adsorption, coagulation–flocculation, ion exchange, oxidation and electrochemical methods (Lin and Peng 1994, 1996). However, the previously mentioned ways for clean-up prove to be quite expensive, limiting their application at large-scale performances (Moreira et al. 2000). Dye decolorization is also achieved by routine anaerobic treatment of the effluents; nevertheless, reduction of azo dyes (up to 50 % of the total amount of dyes used in the textile industry) by the bacterial reductases produces uncolored, highly toxic aromatic amines.

Laccases (benzenediol:oxygen oxidoreductases, EC 1.10.3.2) are blue multicopper oxidases that catalyze the oxidation of an array of aromatic substrates concomitantly with the reduction of molecular oxygen to water (Giardina et al. 2010; Shujing et al. 2013). Fungal laccases have many advantages, such as substrate non-specific, directly oxidizing various phenolic compounds, using molecular oxygen as the final electron acceptor instead of hydrogen peroxide, showing a considerable level of stability in the extracellular environment. Therefore, fungal laccases have been widely applied in biotechnology and industry, such as delignification of lignocellulosics, paper pulping/bleaching, and degradation of different recalcitrant compounds, bioremediation, sewage treatment, dye decolorization and biosensors (Shujing et al. 2013; Osma et al. 2010; Shervedani and Amini 2012).

Therefore, in the present study, a laccase from novel white-rot fungus, identified as *Marasmius* sp. BBKAV79 was subjected to purification, and purified laccase was applied for decolorization of the textile dyes.

## Materials and methods

### Chemicals

Sephadex G-100, DEAE-cellulose and guaiacol were purchased from Sigma-Aldrich Co, St Louis, USA. Standard protein markers were purchased from Merck, Genei, India. Dyes were collected from local textile industry. All chemicals used were of the highest purity available and of the analytical grade.

### Microorganism

Organism screening for laccase-producing microbes on potato dextrose agar (PDA) plates containing indicators resulted in isolation of eight fungal strains. Isolates showing positive reaction were maintained on PDA plates at 30 °C and stored at 4 °C. The best laccase-producing isolate was identified by 18S ribosomal RNA gene sequence deposited in GenBank database and identified as *Marasmius* sp. BBKAV79 (GenBank Accession Number KP455496, KP455497). This isolate is used for the purification and dye decolorization study.

### Laccase production

Yeast extract peptone dextrose–Copper sulfate (YPD–Cu) medium; glucose 20.0 g/l, peptone 5.0 g l^−l^, yeast extract 2.0 g l^−l^ and copper sulfate 100.0 mg l^−l^ (Adiveppa and Basappa 2015).

#### Extracellular enzyme activity

The laccase activity was assayed at room temperature using 10 mM Guaiacol in 100 mM sodium acetate buffer (pH 5.0). The reaction mixture contained 3.0 ml acetate buffer, 1.0 ml Guaiacol and 1.0 ml enzyme source. The change in the absorbance of the reaction mixture containing guaiacol was monitored at 470 nm for 10 min of incubation using UV Spectrophotometer. Enzyme activity is measured in U ml^−1^ which is defined as the amount of enzyme catalyzing the production of one micromole of colored product per min per ml (Jhadav et al. 2009).

$${\mathbf{Calculation}}:\,{\text{Volume}}\,{\text{activity}}\,({\text{U}}\,{\text{ml}}\,{\text{l}}^{{ - 1}} ) = \frac{{\Delta A470\,{\text{nm}}\,\min \,{\text{l}}^{{ - 1}} \times 4 \times V_{\rm t} \times {\text{dilution}}\,{\text{factor}}}}{{ \EUR\times V_{\rm s} }}$$

where *V*_t_ = final volume of reaction mixture (ml) = 5.0; *V*_s_ = sample volume (ml) = 1.0; $\EUR$ = extinction co-efficient of guaiacol = 6740 M^−1^ cm^−1^; 4 = derived from unit definition and principle.

### Extraction and purification of enzyme

Five-day-old *Marasmius* sp. BBKAV79 culture was used for laccase purification. The *Marasmius sp.* BBKAV79 culture supernatant was initially filtered through cheese cloth to remove mycelial debris. Cells were removed by centrifugation at 10,000 rpm for 10 min at 4 °C. Cell-free supernatant was subjected to 80 % ammonium sulfate salt precipitation, and protein precipitate was resuspended in 0.05 M sodium acetate buffer (pH 5.5) that was dialyzed against 5 mM buffer for 12 h. The sample was dialyzed against a large volume of 0.005 M sodium acetate buffer (pH 5.5) using dialysis membrane supplied by Hi-Media Laboratories, India. Dialyzed product was stored in the refrigerator at 4 °C. The dialyzed protein was subjected to gel filtration chromatography; the dialyzed enzyme fraction (3.0 ml) was loaded onto a Sephadex G-100 column. The active fractions were pooled and used for ion-exchange column chromatography on a DEAE-cellulose column that was pre-equilibrated with sodium acetate buffer (0.05 M, pH 5.5). The protein was eluted (flow rate 60 ml h^–l^) with a linear gradient of NaCl (0.1–1 M) in the same buffer. A total of 40 fractions were collected and assayed for protein and enzyme activity. The purity of the enzyme protein was checked by non-denaturing PAGE.

#### Zymogram analysis of laccase on native-PAGE

Native-PAGE was performed as described by Gabriel (1971). Zymogram analysis for laccase activity was performed after native-PAGE was done, as reported earlier (Das et al. 1997), using 5 mM guaiacol in 10 mM sodium acetate buffer (pH 5.6) at room temperature.

### Characterization of laccase

#### Effect of pH and temperature on laccase activity

The effect of pH on laccase activity was studied using guaiacol as substrate dissolved in different buffers of pH (pH 5–11) and incubated at 40 °C and absorbance was recorded at 470 nm. The buffer systems used were acetate buffer (pH 5–6); sodium acetate buffer (pH 5.5); phosphate buffer (pH 7.2); Tris–HCl buffer (pH 8.2) and glycine–NaOH buffer (pH 9–11).

Effect of temperature can be studied by incubating the enzyme at different temperatures ranges (10–60 °C). After incubation, laccase activity was measured by standard enzyme assay. The effect of temperature on the enzyme stability was investigated by incubating the enzyme solution for 15, 30 and 60 min in a 100-mM sodium acetate buffer (pH 5.5) at 40 °C. After incubation, the remaining activity was determined.

#### Effect of metal ions on laccase activity

To study the effect of various metal ions on enzyme activity, the enzyme was incubated with (20 mM) HgCl_2_, FeSO_4_, AgNO_3_, MnSO_4_, MgSO_4_ and ZnSO_4_ at 40 °C for 10 min. Then, the enzyme assay was done by standard enzyme assay protocol.

#### Effect of inhibitors and additives on laccase activity

Laccase inhibitors were selected to evaluate their effect on the purified laccase. The enzyme was incubated with (20 mM) PMSF, EDTA and 1, 10-phenanthroline at 40 °C, and laccase activity was measured. The effect of additives like H_2_O_2_, SDS and NaCl, on activity of purified enzyme was evaluated, and laccase activity was measured.

#### Kinetic properties

The oxidation of substrates by the purified laccase was determined spectrophotometrically at the specific wavelength of substrate. The assay was performed by measuring the increase at the A_470_ for Guaiacol in a 100-mM sodium acetate buffer (pH 5.5). The reaction rate was determined at the substrate (guaiacol) in the concentration range of 0.18–10 mM. The kinetic constants, *K*_m_ and *V*_max_, of the enzyme were determined using a Lineweaver–Burk plot with guaiacol as the substrate.

### Decolorization of dyes

The purified laccase was used to test its efficiency in decolorization of textile dyes. Assay was carried out by incubating the enzyme with dyes for 6–96 h at room temperature. The final concentration of dye in the medium on day zero was considered as control (Mohammed et al. 2013). Various dyes such as Navy blue HER, Green HE4BD and Orange HE2R were monitored at their absorbance maxima at 620, 640 and 420 nm, respectively (Saratale et al. 2009). The percentage of decolorization achieved was calculated with reference to the control samples that were not treated with the enzyme. Percent (%) of dye decolorization was calculated as the formula:

$$ {\mathbf{Decolorization}}\;\left( \% \right) = \frac{{\text{Initial} \, \text{absorbance}{-}\text{Final} \, \text{absorbance}}}{{\text{Initial absorbance}}} \times \, 100 $$

The effect of dye decolorization was determined by the decrease in absorbance under the maximum wavelength of the dye, respectively. The efficiency of decolorization was expressed in terms of percentage (Saratale et al. 2009).

#### Statistical analysis

Data were analyzed by one-way analysis of variance (ANOVA) followed by Tukey’s HSD post hoc test. Readings were considered significant when *P* was ≤0.05.

## Results and discussion

### Purification and characterization of laccase

**Fig. 1:**
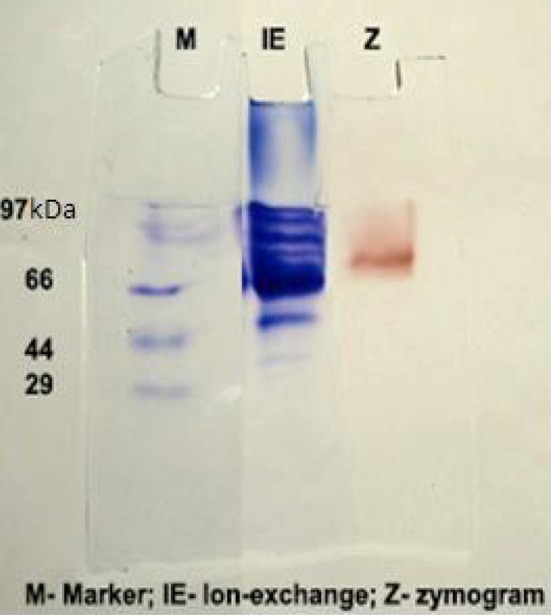
Non-denaturing PAGE for determination of purity and determination of molecular weight of the laccase (molecular weight ~ 75 kDa)

The extracellular laccase from *Marasmius sp.* BBKAV79 was purified to 376.66 total purity with a yield of 13.5 % (Table 1), using a series of purification steps that included ammonium sulfate precipitation, dialyses, gel filtration using Sephadex G-100 column chromatography and DEAE-cellulose using ion-exchange column chromatography. The purified laccase was analyzed by non-denaturing PAGE (Fig. 1). The appearance of a ~75-kDa protein band in native-PAGE indicates that the laccase was purified by this scheme. The relative molecular mass of the purified laccase was found to be ~75 kDa. Zymogram analysis for laccase activity was performed on native-PAGE. Native-PAGE was carried out under non-denaturating conditions. The activity staining of laccase, with guaiacol as substrate, revealed that the single protein band corresponded with activity of the laccase. This result is consistent with most laccases, which are monomeric glycoproteins having a molecular mass of 50–80 kDa (Rosana et al. 2007; Thitinard et al. 2011). Thanunchanok et al. (2014) have reported that the 71 kDa molecular weight of laccase by *Trametes polyzona*. Chaurasia et al. (2013a, b) have showed that the 70 kDa molecular weight of laccase by *Phellinus linteus*. Some species of *Pleurotus* possess a number of different laccase isozymes with molecular masses ranging from 34 to 85 kDa (Palmieri et al. 2003; Pozdnyakova et al. 2006; Wang and Ng 2006b). Mainak and Rintu (2015) have found that the 66 kDa molecular weight of laccase by *Lentinus squarrosulus* MR13. Patel et al. (2014) have reported that the molecular weight of laccase protein was found to be more than 68,420 Da, and zymogram analysis was performed. The purified laccase from *Mycena purpureofusca* appeared as a single band with a molecular weight of 61.7 kDa in SDS-PAGE (Shujing et al. 2013).

**Table 1:** Purification of laccase from *Marasmius* sp. BBKAV79

Purification steps	Activity (U ml^−1^)	Protein concentration (mg ml^−1^)	Specific activity (U mg^−1^)	Yield (%)	Fold purity (%)
Crude	0.962	15.98	0.060	100	1
Dialysis	1.31	9.83	0.133	61.31	221.66
Gel filtration chromatography	0.722	3.78	0.191	23.65	318.6
Ion-exchange chromatography	0.490	2.16	0.226	13.5	376.66

#### Elution of laccase from gel filtration chromatography

**Fig. 2:**
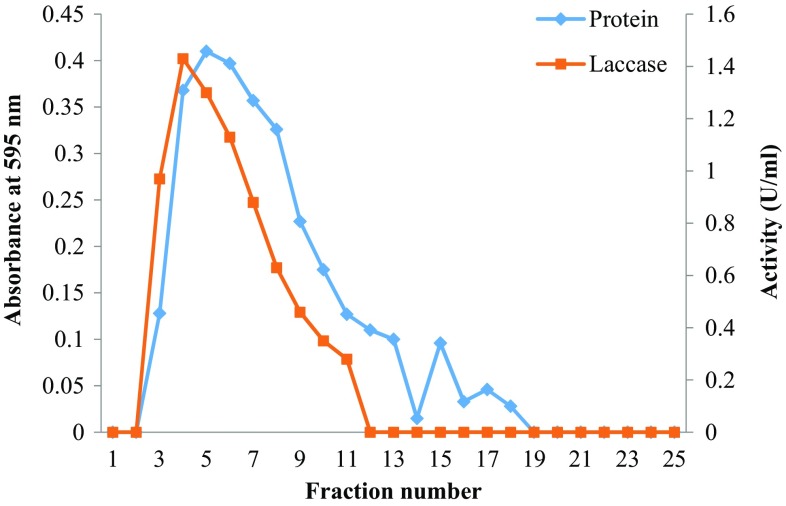
Elution profile of *Marasmius* sp. BBKAV79 laccase from Sephadex G-100 gel filtration column

The laccase loaded on Sephadex has eluted in early fractions (3–13) along with peak in protein. The pooled active fractions were further purified on ion-exchange column (Fig. 2).

#### Laccase elution on ion-exchange chromatography

**Fig. 3:**
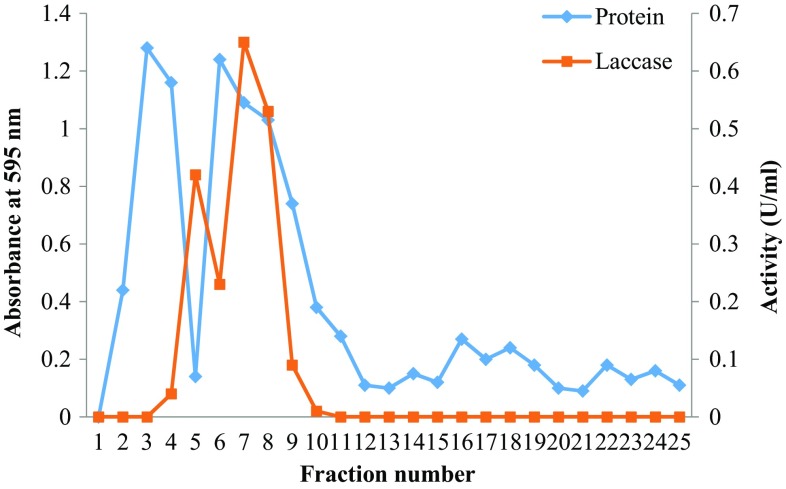
Elution profile of *Marasmius* sp. BBKAV79 laccase from DEAE-Sephadex column

The early elution of laccase was also noted on ion-exchange column along with protein eluted at peak (Fig. 3).

### Characterization of laccase

#### Effect of pH and temperature on laccase activity

**Fig. 4:**
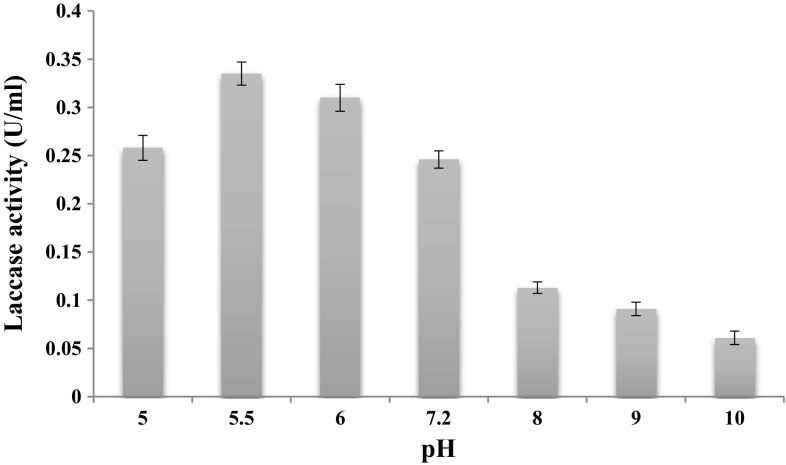
Effect of pH on the activity of the purified laccase

The optimum pH for the purified laccase enzyme was observed in 50 mM sodium acetate with pH 5.5 (0.610 U ml^−1^) followed by pH 6.0 (0.541 U ml^−1^), pH 7.2 (0.489 U ml^−1^), pH 5.0 (0.423 U ml^−1^), pH 8.0 (0.368 U ml^−1^), pH 9.0 (0.345 U ml^−1^), pH 10.0 (0.295 U ml^−1^) and pH 11.0 (0.278 U ml^−1^) (Fig. 4), guaiacol as substrate. Most fungal laccases are functional at acidic or neutral pH values but lose their activities under alkaline conditions (Zhang et al. 2009; Zou et al. 2012). As a rule, many fungal laccases exhibit pH optima in the acidic pH range that vary depending on the type of substrate: for ABTS from pH 2–5, for 2, 6-dimethoxyphenol in the range of pH 3–8 and for syringaldazine between pH 3.5 and 7.0 (Baldrian 2006). Muhammad et al. (2012) have reported the maximum laccase activity at pH 5.0. Chaurasia et al. (2013a, b) have reported the maximum laccase activity at pH 5.0.

**Fig. 5:**
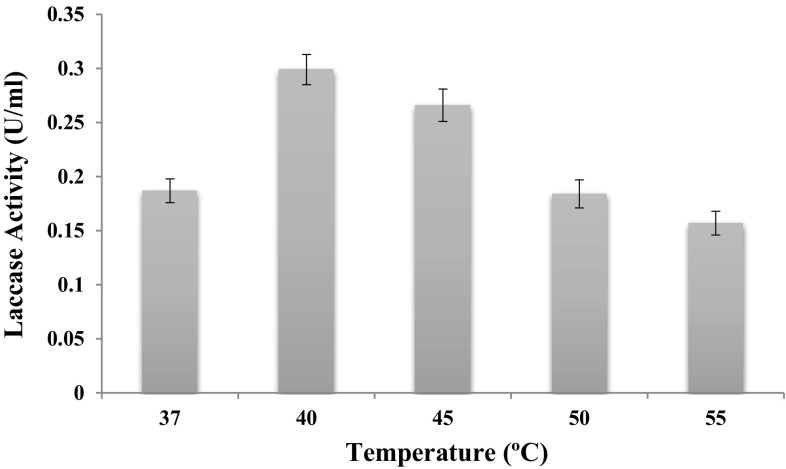
Effect of temperature on the activity of purified laccase

The temperature of activity for this purified laccase was found to be optimum at 40 °C (0.740 U ml^−1^) followed by 50 °C (0.665 U ml^−1^), 55 °C (0.590 U ml^−1^), 60 °C (0.445 U ml^−1^) and room temperature (RT)  °C (0.227 U ml^−1^) (Fig. 5), similar to other laccases from *Lentinus tigrinus* (60 °C) (Xu and Wang 2012), *Tricholoma matsutake* (60 °C) (Lijing et al. 2015), *Ganoderma lucidum* (60 °C) (Wang and Ng 2006a), *Clitocybe maxima* (60 °C) (Zhang et al. 2010), *Hericium erinaceus* (40 °C) (Wang and Ng 2004) and *Lentinula edodes* (40 °C) (Nagai et al. 2002). Chaurasia et al. (2013a, b) have reported the maximum laccase activity at 40 °C. On the other hand, *Tricarpelema giganteum* (Hyoung et al. 2004), *Lentinula edodes* (Sun et al. 2011), *Pleurotus eryngii* (Wang and Ng 2006b), and *Ganoderma lucidum* (Wang and Ng 2006a) laccases demonstrated a higher optimal temperature of 70 °C. Farnet et al. (2000) described a laccase from *Marasmius quercophilus*, a white-rot fungus, with temperature optimum at 80 °C. Shujing et al. (2013) have reported optimum temperature at 50 °C. Muhammad et al. (2012) have reported the maximum laccase activity at 40 °C. Guo-Qing et al. (2010) have showed that the maximum activity of laccase was observed at 60 °C. Thermal stability of laccase was investigated by measurement of the stability activity after incubation of purified laccase in 50 mM sodium acetate buffer, pH 5.5 at 40 °C for 15, 30 and 60 min. Laccase activity was stable up to 15 min, but activity was lost at 30 and 60 min. Laccase activity about 85.32, 80.69 and 75.7 % and lost about 14.68, 19.31 and 24.3 % at 15, 30 and 60 min, respectively.

#### Effect of metal ions on laccase activity

The stability of enzyme activity against different metal ions was studied, and it was observed that HgCl_2_ and AgNO_3_ completely inhibited the enzyme activity followed by 40 % enzyme activity inhibited by FeSO_4_ (Table 2). Addition of Fe^2+^ ions caused the disappearance of most activity; negative effect of ferrous ion in low concentrations was demonstrated in previous studies (Ben and Sayadi 2011; Daâssi et al. 2013; Murugesan et al. 2009). Mainak and Rintu (2015) have showed that Na_2_S has completely inhibited the enzyme activity. More et al. (2011) have reported that the zinc inactivated the enzyme completely at 2 mM concentration. The observations indicated that the effect of metal ions on laccase activity was highly dependent on its source and the type of metals used, which had a great influence on the catalytic activity of the enzyme.

**Table 2:** Effect of metal ions on the activity of the purified laccase from *Marasmius* sp. BBKAV79

Metal ions (20 mM)	Residual activity (%)
FeSO_4_	58.3
AgNO_3_	3.4
MnSO_4_	89.36
MgSO_4_	82.85
HgCl_2_	3.5
ZnSO_4_	92.85

#### Effect of inhibitors and additives on laccase activity

The activity of laccases is inhibited by various organic and inorganic compounds (Morozova et al. 2007). Anions such as the halides, azide, cyanide and hydroxide bind to the type 2 and type 3 copper atoms of laccases, which disrupts the electron transfer system, resulting in enzyme inhibition (Gianfreda et al. 1999). Three potential inhibitors (PMSF, EDTA and 1, 10-phenanthroline ) were evaluated to check the inhibition properties of laccase. It was observed that PMSF inhibits the little enzyme activity (Table 3). This is non-serine and non-metallo laccase. Mainak and Rintu (2015) have reported that the dithiothreitol acted as a potent inhibitor which was able to inhibit the enzyme completely at 0.1 mM concentration. More et al. (2011) have reported that the sodium azide was a potent inhibitor of enzyme, which inactivated the purified laccase completely. Chakroun et al. (2010) have reported that the *Trichoderma atroviride* laccase was strongly inhibited by the typical laccase inhibitor sodium azide, but it was not sensitive to EDTA and SDS. Sadhasivam et al. (2008) have reported that the *Trichoderma harzianum* laccase was mildly inhibited by the metal chelator EDTA at 1 mM concentration (16.8 % inhibition).

**Table 3:** Effect of inhibitor and additives on the activity of the purified laccase from *Marasmius* sp. BBKAV79

Inhibitor (20 mM)	Residual activity (%)
PMSF	91.26
EDTA	110.15
1, 10-phenanthroline	105.07
Additives	
1 % SDS	33.17
1 % H_2_O_2_	1.49
1 % NaCl	23.25

The effect of additives like H_2_O_2_, SDS and NaCl, on activity of purified enzyme was evaluated. Results exhibited that H_2_O_2_ inhibited the enzyme activity followed by NaCl and SDS (Table 3). Laccase has not shown tolerance to bleaching agent, and is also not tolerant to detergent and salt. Mainak and Rintu (2015) have reported that, at 10 mM concentration of SDS, the enzyme was completely inhibited.

#### Kinetic properties

**Fig. 6:**
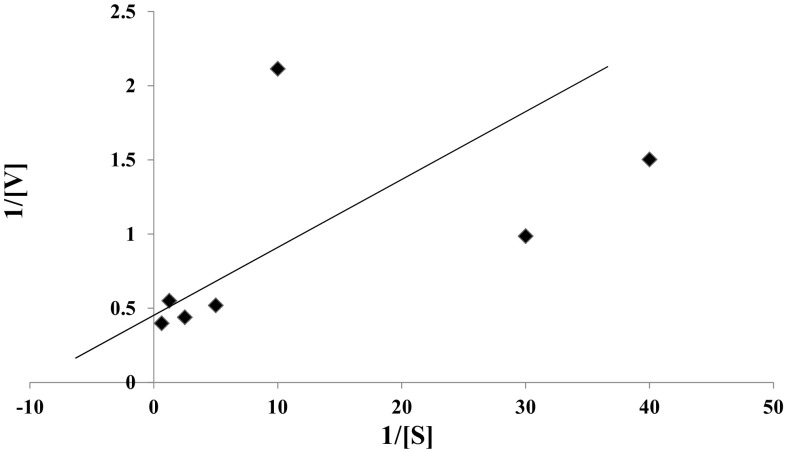
Lineweaver–Burk plot with guaiacol as substrate

The kinetic parameters *K*_m_ and *V*_max_ of purified laccase from *Marasmius* sp. BBKAV79 were found to be 3.03 mM and 5 μmol min^−1^, respectively (Fig. 6). Mainak and Rintu (2015) have showed that the *K*_m_ and *V*_max_ values of the purified yellow laccase were 0.0714 mM and 0.0091 mM min^−1^. Moon-Jeong et al. (2015) have reported that the *K*_m_ value of the enzyme for substrate ABTS is 12.8 µM, and its corresponding Vmax value is 8125.4 U mg^−1^. The laccase in *Trametes* sp. strain AH28-2 had a *K*_m_ value of 25 μM for ABTS (Xiao et al. 2003). Shujing et al. (2013) have reported that the *K*_m_ and Vmax values of the purified laccase were 0.296 mM and 0.0645 mM min^−1^, respectively, with ABTS as substrate. When guaiacol was used as a substrate, the purified laccases of *Fusarium solani* MAS2 (Wu et al. 2010) and *Trichoderma harzianum* WL1 (Sadhasivam et al. 2008) showed *K*_m_ values of 10.23 and 2.66 mM, respectively. Thanunchanok et al. (2014) have reported that the *K*_m_ and *V*_max_ values of the purified laccase were 0.15 mM and 1.84 mM min^−1^, respectively.

### Decolorization of textile dyes

**Fig. 7:**
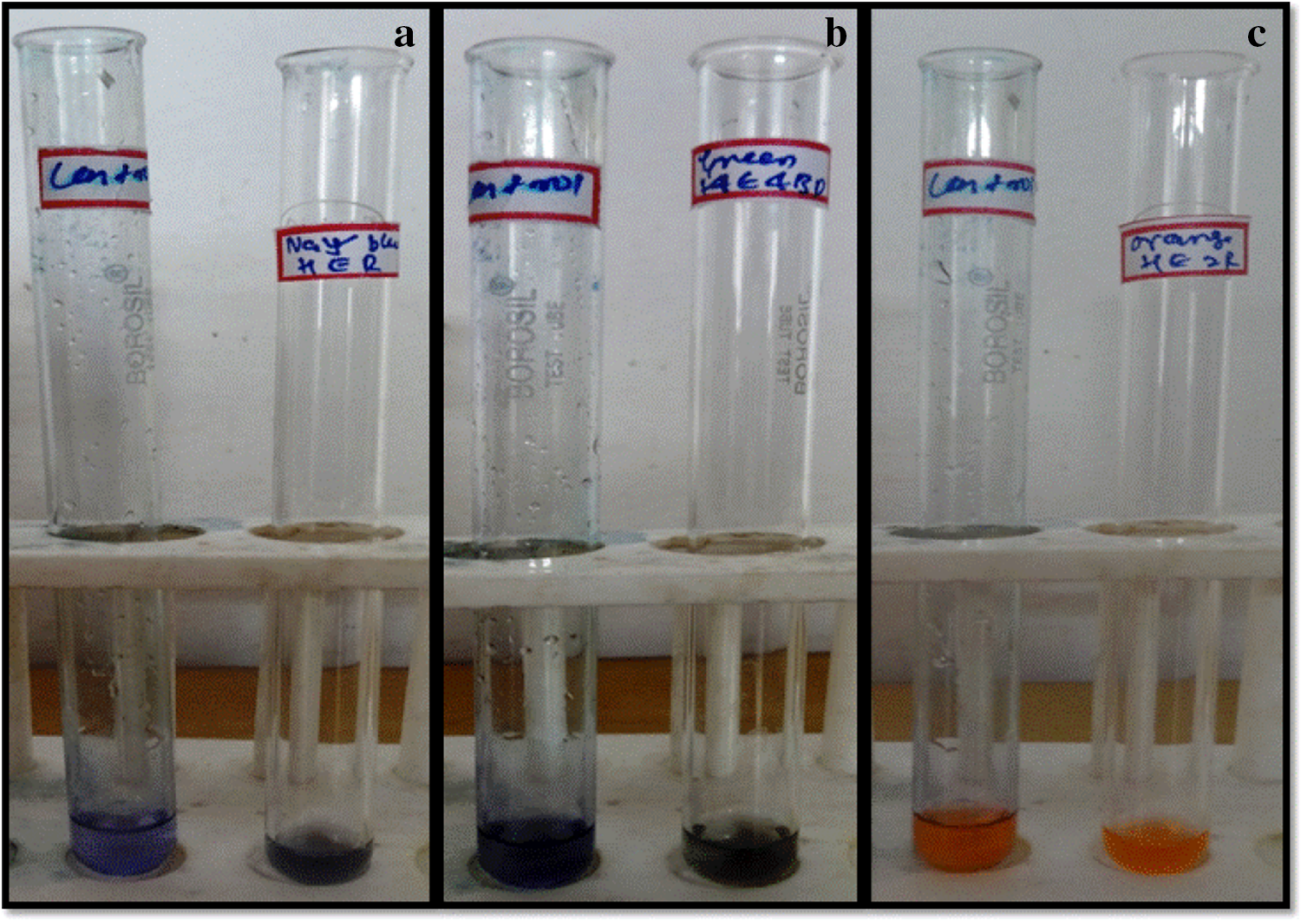
Decolorization of dyes using purified laccase **a** *Orange* HE2R, **b** *Navy blue* HER, **c** *Green* HE4BD

Textile industries consume large volumes of water and chemicals for wet processing of textiles. The presence of very low concentrations of dyes in effluents is highly visible and undesirable (Nigam et al. 2000). Due to their chemical structure, dyes are resistant to fading on exposure to light, water, and many chemicals, and decolorization of textile dye effluents does not occur when they are treated aerobically by sewerage systems (Gisele et al. 2013). Enzyme-based decolorization is an efficient method and of current interest in industrial effluent treatment (Abadulla et al. 2000b). Laccase-mediated azo dye decolorization has been described with crude and purified forms from many fungi; however, most of the laccases required redox mediators (Abadulla et al. 2000a; Zille et al. 2003; Baldrian 2004). In our study, *Marasmius* sp. BBKAV79 and purified laccase was investigated for their ability to decolorize three textile dyes, without any additional redox mediator, namely Navy blue HER, Green HE4BD and Orange HE2R, which were decolorized by 72–76 % within 96 h when treated with *Marasmius* sp. BBKAV79 (Tables 4, 6). On the other hand, the synthetic dyes (0.02 %) were decolorized by 88–93 % within 96 h when treated with purified laccase (Fig. 7; Tables 5, 7). Mohammed et al. (2013) have tested the *Pseudomonas putida* for their dye decolorizing ability against synthetic dyes and industrial effluents. Moorthi et al. (2007) have tested the white-rot fungi *Trametes hirsute* and *Pleurotus florida* for their dye decolorizing ability against reactive dyes Blue CA, Black B133 and Corazol violet SR. It has been reported that extracellular enzymes produced by *Pleurotus sajor*-*caju* under suspension culture completely decolorized several phenolic azo dyes including AR 18 at 50 mg l^−l^ concentrations, and that most of the dye decolorization activity was mainly due to laccase (Chagas and Durrant 2001).

**Table 4:** Decolorization of textile dyes by *Marasmius* sp. BBKAV79

Dye	Decolorization (%)				
	6 h	12 h	24 h	48 h	96 h
Navy blue HER	4.01	22.03	43.68	64.54	72.47
Green HE4BD	13.38	30.19	48.37	59.31	75.31
Orange HE2R	17.03	38.12	58.10	76.42	76.58

**Table 5:** Decolorization of textile dyes by purified laccase

Dye	Decolorization (%)				
	6 h	12 h	24 h	48 h	96 h
Navy blue HER	30.63	54.65	66.56	79.94	93.90
Green HE4BD	21.90	39.17	65.28	76.03	90.24
Orange HE2R	17.03	38.12	58.10	76.42	88.54

**Table 6:** Result of decolorization of textile dyes by *Marasmius* sp. BBKAV79

Dye	0 h	6 h	12 h	24 h	48 h	96 h	*F* and *P* values
Navy blue HER	1.02 ± 0.07	0.98 ± 0.05	0.79 ± 0.07	0.57 ± 0.01	0.36 ± 0.02	0.28 ± 0.02	*F*_5,12_ = 640.7, *P* = 0.00
Green HE4BD	1.10 ± 0.01	0.95 ± 0.01	0.77 ± 0.01	0.57 ± 0.01	0.44 ± 0.03	0.27 ± 0.08	*F*_5,12_ = 969.6, *P* = 0.00
Orange HE2R	1.10 ± 0.01	0.96 ± 0.01	0.74 ± 0.01	0.59 ± 0.09	0.38 ± 0.01	0.25 ± 0.04	*F*_5,12_ = 1316.0, *P* = 0.00

If *P* value is ≤ 0.05 (*P* ≤0.05), then it is significantly different from other durations, and if *P* value is > 0.05 (*P* >0.05), then it is not significant

**Table 7:** Result of decolorization of textile dyes by purified laccase

Dye	0 h	6 h	12 h	24 h	48 h	96 h	*F* and *P* values
Navy blue HER	1.72 ± 0.09	1.18 ± 0.02	0.79 ± 0.08	0.57 ± 0.02	0.34 ± 0.02	0.10 ± 0.01	*F*_5,12_ = 1158.0, *P* = 0.00
Green HE4BD	1.21 ± 0.01	0.94 ± 0.00	0.74 ± 0.03	0.42 ± 0.02	0.29 ± 0.06	0.11 ± 0.02	*F*_5,12_ = 1605.0, *P* = 0.00
Orange HE2R	1.09 ± 0.03	0.90 ± 0.04	0.68 ± 0.04	0.45 ± 0.02	0.26 ± 0.02	0.12 ± 0.02	*F*_5,12_ = 1182.0, *P* = 0.00

If *P* value is ≤ 0.05 (*P* ≤0.05), then it is significantly different from other hours, and if *P* value is > 0.05 (*P* >0.05), then it is not significant

## Conclusion

The laccase enzyme isolated and purified from *Marasmius* sp. BBKAV79 demonstrated maximum activity and stability at 40 °C and pH 5.5, respectively. The textile dyes were found to be decolorized up to 72–76 % and 88–93 % upon the media containing respective dye was inoculated with *Marasmius* sp. BBKAV79 at specific durations. Further, the purified laccase, isolated from the *Marasmius* sp. BBKAV79 was also found to decolorize the same in the absence of the organism. Therefore, the use of organism *Marasmius* sp. BBKAV79 and enzyme laccase, isolated from it could prove to be a valuable measure to reduce the magnitude of risk associated with effective decolorization process of textile dyes that, otherwise, could significantly contribute in elevating chemical pollution levels. The use of laccase and *Marasmius* sp. BBKAV79 for decolorization of textile dyes could, hence, be considered as a potential measure by decision-makers for reducing the burden of chemical flow as contributed by dyes, further emphasizing its use in detoxifying the effluents from industries like pulp and paper, textile, paint and electroplating industries, with its further application in wastewater treatment procedures as well.

## References

[CR1] Abadulla E, Robra KH, Gubitz GM, Silva L, Cavaco-Paulo A (2000). Enzymatic decolorization of textile dyeing effluents. Tex Res J..

[CR2] Abadulla E, Tzanov T, Costa S, Robra KH, Cavaco-Paulo A, Gubitz GM (2000). Decolorization and detoxification of textile dyes with a laccase from *Trametes hirsute*. Appl Environ Microbiol.

[CR3] Adiveppa BV, Basappa BK (2015). Isolation, screening and identification of laccase producing fungi. Int J Pharm Biol Sci.

[CR4] Baldrian P (2004). Purification and characterization of laccase from the white-rot fungus *Daedalea quercina* and decolorization of synthetic dyes by the enzyme. Appl Microbiol Biotechnol.

[CR5] Baldrian P (2006). Fungal laccases: occurrence and properties. FEMS Microbiol Rev.

[CR6] Ben YS, Sayadi S (2011). Purification and characterization of a novel trimeric and thermotolerant laccase produced from the ascomycete *Scytalidium thermophilum* strain. J Mol Catal B Enzym.

[CR7] Chagas EP, Durrant LR (2001). Decolorization of azo dyes by *Phanerochaete chrysosporium* and *Pleurotus sajor*-*caju*. Enzym Microbiol Technol.

[CR8] Chakroun H, Mechichi T, Martinez MJ, Dhouib A, Sayadi S (2010). Purification and characterization of a novel laccase from the ascomycete *Trichoderma atroviride*: application on bioremediation of phenolic compounds. Process Biochem.

[CR9] Chaurasia PK, Yadav A, Yadav RSS, Yadava S (2013). Purification and characterization of laccase secreted by *Phellinus linteus* MTCC1175 and its role in the selective oxidation of aromatic methyl group 1. Appl Biochem Microbiol.

[CR10] Chaurasia PK, Yadav A, Yadav RSS, Yadava S (2013). Purification and characterization of laccase from *Coriolopsis floccose* MTCC-1177 and its use in the selective oxidation of aromatic methyl group to aldehyde without mediators. J Chem Sci.

[CR11] Das N, Sengupta S, Mukherjee M (1997). Importance of laccase in vegetative growth of *Pleurotus florida*. Appl Environ Microbiol.

[CR12] David M, Kartheek RM (2015). In vivo studies on hepato-renal impairments in freshwater fish *Cyprinus carpio* following exposure to sublethal concentrations of sodium cyanide. Environ Sci Pollut Res.

[CR13] Farnet AM, Criquet S, Tagger S, Gil G, Le Petit J (2000). Purification, partial characterization, and reactivity with aromatic compounds of two laccases from *Marasmius quercophilus* strain 17. Can J Microbiol.

[CR14] Gabriel O (1971). Locating enzymes on gels. Methods Enzymol.

[CR15] Gianfreda L, Xu F, Bollag JM (1999). Laccases: a useful group of oxidoreductases enzymes. Biorem J.

[CR16] Giardina P, Faraco V, Pezzella C, Piscitelli A, Vanhulle S, Sannia G (2010). Laccases: a never-ending story. Cell Mol Life Sci.

[CR17] Gisele CDSB, Daniela FDS, Rafael C, Roselene FO, Adelar B, Rosane MP (2013). Production of laccase and manganese peroxidase by *Pleurotus pulmonarius* in solid-state cultures and application in dye decolorization. Folia Microbiol.

[CR18] Guo-Qing Z, Yi-Fan W, Xiao-Qing Z, Tzi BN, He-Xiang W (2010). Purification and characterization of a novel laccase from the edible mushroom *Clitocybe maxima*. Process Biochem.

[CR20] Houria G, Oualid H (2009). Degradation of acid blue 25 in aqueous media using 1700 kHz ultrasonic irradiation: ultrasound/Fe(II) and ultrasound/H2O2 combinations. Ultrason Sonochem.

[CR21] Hyoung LD, Ho KJ, Sik PJ, Jun CY, Soo LJ (2004). Isolation and characterization of a novel angiotensin I-converting enzyme inhibitory peptide derived from the edible mushroom *Tricholoma giganteum*. Peptides.

[CR22] Jhadav A, Vamsi KK, Khairnar Y, Boraste A, Gupta N, Trivedi S, Patil P, Gupta G, Gupta M, Mujapara AK, Joshi B, Mishra D (2009). Optimization of production and partial purification of laccase by *Phanerochaete chrysosporium* using submerged fermentation. Int J Microbiol Res.

[CR23] Lijing X, Mengjuan Z, Xiao C, Hexiang W, Guoqing Z (2015) Novel laccase from fresh fruiting bodies of the wild medicinal mushroom *Tricholoma matsutake*. Acta Biochimic Pol 62(1):35–40 10.18388/abp.2014_713 25781157

[CR24] Lin SH, Peng CF (1994). Treatment of textile wastewater by electrochemical methods. Water Res.

[CR25] Lin SH, Peng CF (1996). Continuous treatment of textile wastewater by combined coagulation, electrochemical oxidation and activated sludge. Water Res.

[CR26] Mainak M, Rintu B (2015). Purification and biochemical characterization of a newly produced yellow laccase from *Lentinus squarrosulus* MR13. 3. Biotechnology.

[CR27] Malaja E, von der Ohea PC, Groted M, Kühnee R, Cédric PM, Usseglio-Polaterag P, Bracka W, Schäfer RB (2014). Organic chemicals jeopardize the health of freshwater ecosystems on the continental scale. Proc Natl Acad Sci.

[CR28] Mohammed K, Babu J, Pramod WR (2013). Production of laccase from newly isolated *Pseudomonas putida* and its application in bioremediation of synthetic dyes and industrial effluents. Biocatal Agricult Biotechnol.

[CR01] Moon-Jeong H, Hyoung-Tae C, Hong-Gyu S (2005) Purification and Characterization of Laccase from the White Rot Fungus Trametes versicolor. J Microbiol 43(6):555–560 16410773

[CR29] Moorthi PS, Periyar SS, Sasikalaveni A, Murugesan K, Kalaichelvan PT (2007). Decolorization of textile dyes and their effluents using white rot fungi. Afr J Biotechnol.

[CR30] More SS, Renuka PS, Pruthvi K, Swetha M, Malini S, Veena SM (2011) Isolation, purification, and characterization of fungal laccase from *Pleurotus sp.* SAGE-Hindawi Access to Research Enzyme Research. 2011, Article ID 248735, p 7 10.4061/2011/248735 PMC3184503 21977312

[CR31] Moreira MT, Mielgo I, Feijoo G, Lema JM (2000). Evaluation of different fungal strains in the decolorization of synthetic dyes. Biotechnol Lett.

[CR32] Morozova OV, Shumakovich GP, Gorbacheva MA, Shleev SV, Yaropolov AI (2007). Blue laccases. J Basic Microbiol.

[CR33] Muhammad SA, Aisha A, Zahoor QS, Iram G, Muhammad AA (2012). Identification, purification and characterization of a novel extracellular laccase from *Cladosporium Cladosporioides*. Biotechnol Biotechnol Equip.

[CR34] Murugesan K, Kim YM, Jeon JR, Chang YS (2009). Effect of metal ions on reactive dye decolorization by laccase from *Ganoderma lucidum*. J Hazard Mater.

[CR35] Nagai M, Sato T, Watanabe H, Saito K, Kawata M, Enei H (2002). Purification and characterization of an extracellular laccase from the edible mushroom *Lentinula edodes*, and decolorization of chemically different dyes. Appl Microbiol Biotechnol.

[CR36] Nigam P, Armour G, Banat IM, Singh D, Marchant R (2000). Physical removal of textile dyes and solid state fermentation of dye adsorbed agricultural residues. Bioresour Technol.

[CR37] Osma JF, Toca-Herrera JL, Rodriguez-Couto S (2010). Transformation pathway of Remazol Brilliant Blue R by immobilised laccase. Bioresour Technol.

[CR38] Palmieri G, Cennamo G, Faraco V, Amoresano A, Sannia G, Giardina P (2003). A typical laccase isoenzymes from copper supplemented *Pleurotus ostreatus* cultures. Enzyme Microb Technol.

[CR39] Patel H, Gupte S, Gahlout M, Gupte A (2014). Purification and characterization of an extracellular laccase from solid-state culture of *Pleurotus ostreatus* HP-1. 3. Biotechnology.

[CR40] Pozdnyakova NN, Turkovskaya OV, Yudina EN, Rodakiewicz-Nowak Y (2006). Yellow laccase from the fungus *Pleurotus ostreatus* D1: purification and characterization. Appl Microbiol Biotechnol.

[CR41] Rosana CM, Marcio AM, Jose AS, Carmen VF (2007). Purification, characterization and application of laccase from *Trametes versicolor* for colour and phenolic removal of olive mill wastewater in the presence of 1- hydroxybenzotriazole. Afr J Biotechnol.

[CR42] Sadhasivam S, Savitha S, Swaminathan K, Feng-Huei L (2008). Production, purification and characterization of mid-redox potential laccase from a newly isolated *Trichoderma harzianum* WL1. Process Biochem.

[CR43] Saratale RG, Saratale GD, Chang JS, Govindwar SP (2009). Decolorization and biodegradation of textile dye Navy blue HER by *Trichosporon beigelii* NCIM-3326. J Hazard Mater.

[CR45] Shervedani RK, Amini A (2012). Direct electrochemistry of dopamine on gold- *Agaricus bisporus* laccase enzyme electrode: characterization and quantitative detection. Bioelectrochem.

[CR46] Shujing S, Yonghui Z, Youxiong Q, Bixian L, Kaihui H, Liping X (2013). Purification and characterization of fungal laccase from *Mycena purpureofusca*. Chiang Mai J Sci.

[CR47] Sun J, Wang H, Ng T (2011). Isolation of a laccase with HIV-1 reverse transcriptase inhibitory activity from fresh fruiting bodies of the *Lentinus edodes* (Shiitake mushroom). Indian J Biochem Biophys.

[CR48] Thanunchanok C, Thitinard N, Akira W, Yasuhiko A, Chartchai K, Saisamorn L (2014). Purification and characterization of the extracellular laccase produced by *Trametes polyzona* WR710–1 under solid-state fermentation. J Basic Microbiol.

[CR49] Thitinard N, Watanabe A, Asada Y (2011). Extracellular laccase produced by an edible basidiomycetous mushroom, *Grifola frondosa*: purification and characterization. Biosci Biotechnol Biochem.

[CR50] Vinodhkumar T, Thiripurasundari N, Ramanathan G, Karthik G (2013). Screening of dye degrading bacteria from textile effluents. IJRRPAS.

[CR52] Wang HX, Ng TB (2004). A new laccase from dried fruiting bodies of the monkey head mushroom *Hericium erinaceum*. Biochem Biophys Res Commun.

[CR53] Wang HX, Ng TB (2006). A laccase from the medicinal mushroom *Ganoderma lucidum*. Appl Microbiol Biotechnol.

[CR54] Wang HX, Ng TB (2006). Purification of a laccase from fruiting bodies of the mushroom *Pleurotus eryngii*. Appl Microbiol Biotechnol.

[CR55] Willmott N, Guthrie J, Nelson G (1998). The biotechnology approach to color removal from textile effluent. J Soc Dyes Colour.

[CR56] Wu YR, Luo ZH, Chow RK, Vrijmoed LL (2010). Purification and characterization of an extracellular laccase from the anthracene degrading fungus *Fusarium solani* MAS2. Bioresour Technol.

[CR02] Xiao Y, Tu X, Wang J, Zhang M, Cheng Q, Zeng W, Shi Y (2003) Purification, molecular characterization and reactivity with aromatic compounds of a laccase from basidiomycete *Trametes* sp. strain AH28-2. Appl Microbiol Biotechnol 60(6):700–707 10.1007/s00253-002-1169-3 12664149

[CR57] Xu L, Wang H, Ng T (2012). A laccase with HIV-1 reverse transcriptase inhibitory activity from the broth of mycelial culture of the mushroom *Lentinus tigrinus*. J Biomed Biotechnol.

[CR58] Zhang H, Zhang Y, Huang F, Gao P, Chen J (2009). Purification and characterization of a thermostable laccase with unique oxidative characteristics from *Trametes hirsuta*. Biotechnol Lett.

[CR59] Zhang GQ, Wangl YF, Zhang XQ, Ng TB, Wang HX (2010). Purification and characterization of a novel laccase from the edible mushroom *Clitocybe maxima*. Process Biochem.

[CR60] Zille A, Tzanov T, Gübitz GM, Cavaco-Paulo M (2003). Immobilized laccase for decolourization of reactive black 5 dyeing effluent. Biotechnol Lett.

[CR61] Zou YJ, Wang HX, Ng TB, Huang CY, Zhang JX (2012). Purification and characterization of a novel laccase from the edible mushroom *Hericium coralloides*. J Microbiol.

